# Inhibition of Human and Yeast 20S Proteasome by Analogues of Trypsin Inhibitor SFTI-1

**DOI:** 10.1371/journal.pone.0089465

**Published:** 2014-02-25

**Authors:** Dawid Dębowski, Michał Pikuła, Marta Lubos, Paulina Langa, Piotr Trzonkowski, Adam Lesner, Anna Łęgowska, Krzysztof Rolka

**Affiliations:** 1 Department of Bioorganic Chemistry, Faculty of Chemistry, University of Gdansk, Gdansk, Poland; 2 Department of Clinical Immunology and Transplantology, Medical University of Gdansk, Gdansk, Poland; Florida State University, United States of America

## Abstract

Starting from the primary structure of sunflower trypsin inhibitor SFTI-1, we designed novel non-covalent inhibitors of human and yeast 20S proteasomes. Peptides with Arg residue in P_1_ position and two basic amino acid residues (Lys or/and Arg) in P_2_′ and P_3_′ positions strongly inhibited chymotrypsin-like and caspase-like activities, while trypsin-like activity was poorly modified. We found that some SFTI-1 analogues up-regulated exclusively the chymotrypsin-like activity of latent yeast 20S proteasome.

## Introduction

**Figure 1:**
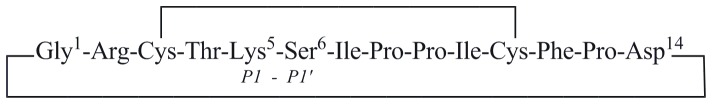
Like other canonical inhibitors, SFTI-1 interacts with a cognate enzyme (i.e. with trypsin) *via* its solvent-exposed binding loop between Cys^3^ and Cys^11^. The central peptide bond between Lys^5^ (P_1_) and Ser^6^ (P_1_′) is known as a reactive site [17]. The adjacent residues placed on the left side of this bond are marked with non-prime P (P_2_, P_3_, … P_n_ etc), whereas, on the right side, with prime P (P_2_′, P_3_′, … P_n_ etc) [18]. Corresponding enzyme substrate binding pockets are called non-prime (S_1_, S_2_, S_3_, …, S_n_) and prime (S_1_′, S_2_′, S_3_′, …,S_n_′) sites, respectively.

The ubiquitin–proteasome system is the major protein degradation pathway in every cell [1], [2]. The eukaryotic proteasome is a potential target for antitumor drugs [3]. To date, two proteasome inhibitors, the Bortezomib (a dipeptide boronic acid) and Carfilzomib (a tetrapeptide epoxyketone), have been approved for the treatment of multiple myeloma [4], [5]. The 26S proteasome is a large multi-enzymatic complex composed of a tube-shaped 20S core particle (CP) capped on one or two ends by 19S regulatory particles [6], [7]. The CP consists of four stacked rings. The internal rings are composed of seven distinct *β* subunits, while the outer rings are composed of seven *α* subunits. The mammalian constitutive CP possesses three pairs of distinct catalytic sites: chymotrypsin-like (ChT-L, *β*5 subunit), trypsin-like (T-L, *β*2) and caspase-like (C-L, *β*1). An alternative form, immunoproteasome, is generated in response to *γ*-interferon stimulation and plays a crucial role in the production of the antigenic peptides presented by major histocompatibility complex class I (MHC-I) [8]. The proteasome is classified as the *N*-terminal nucleophile aminohydrolase (threonine proteases), since it cleaves a peptide bond using the hydroxyl group of an *N*-terminal threonine residue in the catalytic site. Many natural and synthetic compounds have been tested regarding their actual and potential inhibitory activities towards proteasomes [9]. Among them, three are well known proteinaceous inhibitors of serine proteases: soybean-derived Bowman Birk inhibitor (BBI), bovine pancreatic trypsin inhibitor (BPTI) and *Momordica charantia* trypsin inhibitor I (MoCTI-I). BBI is composed of 71 amino acids and specifically inhibits ChT-L activity *in vivo* and *in vitro* in both MCF7 breast cancer cells [10] and human osteosarcoma cells (U2OS cells) [11]. It also provides protection against the development of colorectal tumors, induced chemically by 1,2-dimethylhydrazine (DMH) in *Swiss* mice [12]. BPTI is composed of 58 amino acids and is described as a potent inhibitor (*in vitro* and *ex vivo*) of all catalytic sites of rat and porcine 20S proteasomes [13]. BPTI enters the 20S core particle and blocks the catalytic sites stoichiometrically and competitively with the inhibition constant K_i_ of 2 µm (in the case of ChT-L). MoCTI-I is composed of 30 amino acids and is able to reduce the T-L activity [13]. Surprisingly, the smallest plant canonical inhibitor, sunflower trypsin inhibitor SFTI-1 (Fig. 1), consisting of only 14 amino acids, does not cause any inhibition. SFTI-1 belongs to the Bowman-Birk family as the above-mentioned inhibitors and shares exactly the same mechanism of interaction with target enzymes. Theoretically, its small dimension should facilitate penetration into the 20S core particle and subsequent interactions with the active sites. However, it seems that instead of compound size, the presence of basic amino acid residues at P_2_′ and/or P_3_′ positions of the canonical inhibitor binding loop (Fig. 1) play a pivotal role and determine the activity against proteasome [13].

SFTI-1 is a potent and protease-resistant trypsin inhibitor, its association constant K_a_ is 1.1×10^10^ M^−1^ [14] and the inhibitory constant K_i_ is 0.5 nm [15]. Its monocyclic derivative, devoid of a head to tail connection, exhibits a similar activity (K_a_ = 9.9×10^9^ M^−1^) [14]. Due to its small size and well-defined three-dimensional structure, SFTI-1 has been used for the design of inhibitors of trypsin, chymotrypsin, cathepsin G, matriptase, *β*-tryptase, proteinase K and kallikrein-related peptidase [16].

Here, we aimed to prove that SFTI-1 can be regarded as a convenient template for preparation of potent 20S proteasome inhibitors. A set of monocyclic SFTI-1 analogues having only disulfide bridge were designed and synthesized (Table 1). Most of these analogues except for the peptide IX had one or two basic amino acid residues (Lys or Arg). Those basic amino acid residues we located in position P_2_′ and P_3_′ in place of the naturally occurring Ile and Pro (Table 1). The peptides were tested regarding their effect on the human and yeast 20S proteasomes.

**Table 1:** Primary structures of monocyclic SFTI-1 and its analogues, the positions P_2_′ and P_3_′ in SFTI-1 are in bold, (&) – indicates the presence of a disulfide bridge.

No.	Analogue	Primary structure
**I**	SFTI-1	Gly-Arg-Cys(&)-Thr-Lys-Ser**-Ile**-**Pro**-Pro-Ile-Cys(&)-Phe-Pro-Asp
**II**	[Leu^7^,Lys^8^]SFTI-1	Gly-Arg-Cys(&)-Thr-Lys-Ser-Leu-Lys-Pro-Ile-Cys(&)-Phe-Pro-Asp
**III**	[Arg^5^,Leu^7^,Lys^8^]SFTI-1	Gly-Arg-Cys(&)-Thr-Arg-Ser-Leu-Lys-Pro-Ile-Cys(&)-Phe-Pro-Asp
**IV**	[Arg^7^,Ile^8^]SFTI-1	Gly-Arg-Cys(&)-Thr-Lys-Ser-Arg-Ile-Pro-Ile-Cys(&)-Phe-Pro-Asp
**V**	[Arg^5^,Lys^7,8^]SFTI-1	Gly-Arg-Cys(&)-Thr-Arg-Ser-Lys-Lys-Pro-Ile-Cys(&)-Phe-Pro-Asp
**VI**	[Tyr^5^,Lys^7,8^]SFTI-1	Gly-Arg-Cys(&)-Thr-Tyr-Ser-Lys-Lys-Pro-Ile-Cys(&)-Phe-Pro-Asp
**VII**	[Phe^5^,Lys^7,8^]SFTI-1	Gly-Arg-Cys(&)-Thr-Phe-Ser-Lys-Lys-Pro-Ile-Cys(&)-Phe-Pro-Asp
**VIII**	[Ala^5^,Lys^7,8^]SFTI-1	Gly-Arg-Cys(&)-Thr-Ala-Ser-Lys-Lys-Pro-Ile-Cys(&)-Phe-Pro-Asp
**IX**	[Ala^7,8^]SFTI-1	Gly-Arg-Cys(&)-Thr-Lys-Ser-Ala-Ala-Pro-Ile-Cys(&)-Phe-Pro-Asp
**X**	[Lys^7,8^]SFTI-1	Gly-Arg-Cys(&)-Thr-Lys-Ser-Lys-Lys-Pro-Ile-Cys(&)-Phe-Pro-Asp

## Materials and Methods

### Peptide Synthesis

All peptides were synthesized manually *via* the solid phase approach on 2-chlorotrityl chloride resin (Calbiochem/Novabiochem AG, Switzerland) using Fmoc (fluorenyl-9-methoxycarbonyl) chemistry. The protected amino acid derivatives (GL Biochem, Shanghai, China Ltd) were coupled using an equimolar mixture of *N,N*′-diisopropylcarbodiimide (DIC) and 1-hydroxybenzotriazole (HOBt). The *C*-terminal Fmoc-Asp(OtBu)-OH was attached to 2-chlorotrityl chloride resin in the presence of an equimolar amount of *N*,*N*-diisopropylethylamine (DIPEA) in anhydrous dichloromethane (DCM). Removal of the Fmoc group was performed using 20% piperidine in dimethylformamide (DMF) solution with addition of Triton X-100. After completing the syntheses, the peptides were cleaved from the resin using a mixture of trifluoroacetic acid (TFA)/triisopropylsilane/phenol/water (88∶5∶2∶5, v/v/v/v). Disulfide bridge formation was performed using a 0.1 m iodine solution in methanol.

The purity of each peptide was checked by a reverse-phase high-performance liquid chromatography (RP-HPLC) on a Shimadzu Prominence UFLC (Japan, Kyoto) equipped with a Supelco Supelcosil column (250×4.6 mm, 5 µm, C-8, Supelco/Sigma-Aldrich Co., USA) and a UV-Vis detector. The solvent system was 0.1% TFA (A) and 80% acetonitrile in A (B). A linear gradient from 10% to 90% B within 30 min., with a flow rate of 1.0 ml/min and monitoring set at 226 nm. Crude oligomers were purified by RP-HPLC on a Beckman Gold System (Beckman, USA) using semi-preparative Discovery BIO Wide Pore column C-8 (250×10 mm, 5 µm, Supelco/Sigma-Aldrich Co., USA). The solvent system was 0.1% TFA (A) and 80% acetonitrile in A (B). The mass spectrometry analysis of the synthesized compounds was carried out on a MALDI MS (Biflex III MALDI-TOF spectrometer, Bruker Daltonics, Germany) using an *α*-cyano-4-hydroxycinnamic acid (CCA) and/or 2,5-dihydroxybenzoic acid (DHB) matrix.

### Determination of Peptide Concentrations

The molar concentration of each peptide was determined using HPLC method as described previously [19]. Peptides were dissolved in 50 mm Tris-HCl (pH 8.1) buffer at concentration of about 4 mg/ml. Equal volume of each solution was injected into the HPLC column. The peptide peak area was integrated and compared to that of the standardized monocyclic SFTI-1. The stock solutions of monocyclic SFTI-1 (about 2 mg/ml) were prepared with 1 mm HCl_aq_. The concentration of bovine *β*-trypsin (active form) was determined with 4-nitrophenyl 4-guanidinobenzoate hydrochloride solution (NPGB). The standardized trypsin solution (2.0×10^−4^ m) was used to titrate SFTI-1.

### Proteolytic Susceptibility Assays

Analogues **II**, **III**, **IV** and **V** (about 4.0×10^−8^ mol) were incubated with bovine *β*-trypsin (2.0×10^−9^ mol) in 1.5 ml of 100 mm Tris-HCl buffer (pH 8.3) containing 20 mm CaCl_2_ and 0.005% Triton X-100. Five analogues **I**–**V** were incubated with human 20S proteasome, moreover, three of them (**III**, **V**, and **IX)** were incubated with yeast 20S. Both assays with proteasomes were conducted in 50 mm Tris-HCl (pH 8.1) with 0.02% (w/v) sodium dodecyl sulfate (SDS). Incubation was carried out at room temperature and aliquots of the mixture were extracted periodically and subjected to RP-HPLC and MALDI MS analysis.

### Determination of Purified Human and Yeast 20S Activity *in vitro*

The peptidase activities of 20S proteasome were assayed using fluorogenic tri- and tetra-peptides coupled at their *C*-termini with 7-amino-4-methylcoumarin group (AMC). The initial concentration of purified yeast 20S proteasome [E_0_] (the enzyme was kindly gifted by Prof. Michael Groll, proteasome preparation described elsewhere [20]) was about 0.9 nm, whereas purified human erythrocyte 20S (Boston Biochem, USA) was about 2.7 nm. An assay buffer was composed of 50 mm Tris-HCl (pH 8.1) and 0.02% (w/v) SDS (exceptionally, T-L activity was measured in Tris buffer without SDS, that may trigger precipitation of substrates containing the guanidino group [21]). Small amount of SDS stimulates peptide cleavage by latent yeast and human 20S [22]. The SFTI-1 analogues were incubated with 20S proteasome in assay buffer at 37°C for 30 min. Reactions were performed in 96-well black BRAND plates (Germany). The final volume of the solution in each well was 200 µl. The following selective fluorogenic substrates were used: Suc-LLVY-AMC (ChT-L, [S]_0_ = 5.2 µm for yeast 20S and [S]_0_ = 52 µm for human 20S), Z-LLR-AMC (where Z is benzyloxycarbonyl group, T-L, [S]_0_ = 52 µm for human 20S) and Z-LLE-AMC (C-L, [S]_0_ = 79 µm for human 20S). Proteasomal subsite activities were determined by monitoring the AMC liberation over time with a Fluorostar Omega (BMG Labtech, Germany) using excitation and emission wavelengths of 380 nm and 450 nm, respectively. Percentage inhibition of 20S was calculated relative to a control sample without inhibitor. The inhibitory activities of the compounds studied were expressed as IC_50_ values (inhibitor concentrations giving 50% inhibition) calculated from plots of enzyme activity (% of control) versus the inhibitor’s concentration (µm) using a four-parameter fit model (GraFit 5.0.12 Erithacus Software Ltd., Surrey, UK). The inhibition constant values K_i_ of peptide aldehyde Z-LLL-CHO (MG-132, used as positive control, where Z is benzyloxycarbonyl group) as well as of the analogue **V** were measured from the slopes of the reaction progress curves plotted against substrate concentrations (using the GraFit software package). Human 20S concentration was kept constant at 3.57 nm. Substrate concentrations were varied from 10.8 to 173.4 µm. The remaining K_i_ values were calculated using an online IC_50_-to-K_i_ converter tool (BotDB Database [23]), assuming competitive inhibition, according to the following relationship: IC_50_ = K_i_(1+ S/K_m_). Association constants K_a_ were measured as described previously [14] and calculated using a two-parameter fit model (using the GraFit software package).

## Results

**Figure 2:**
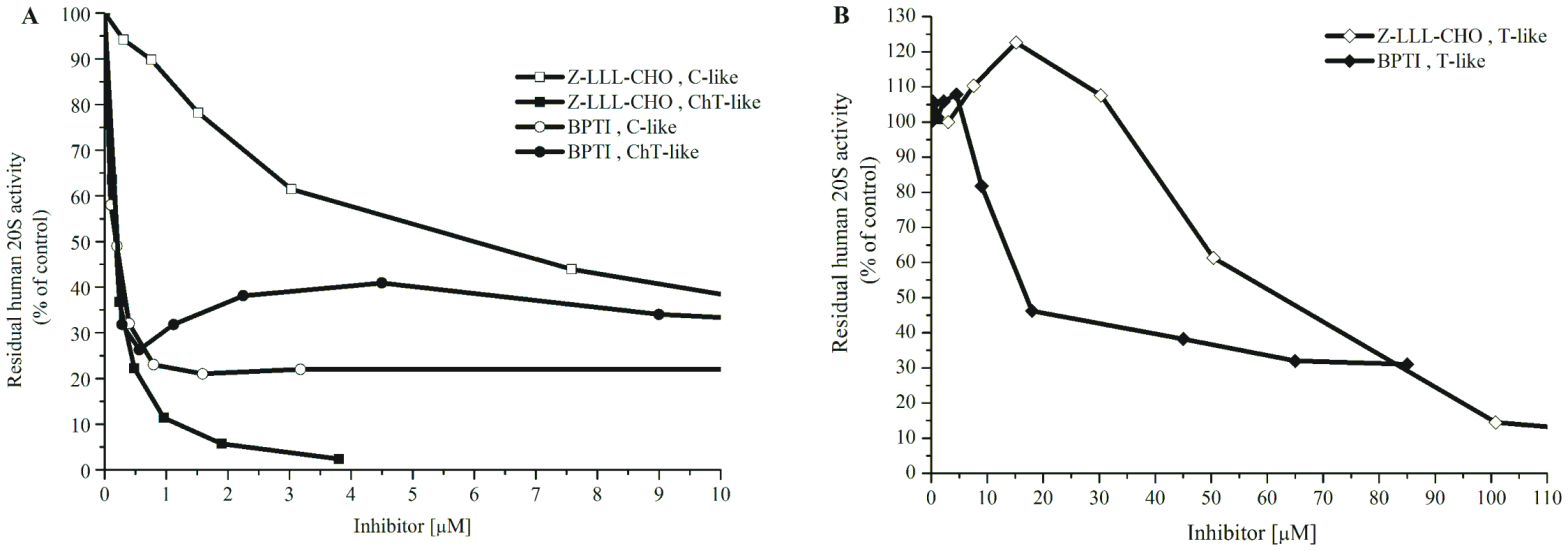
Inhibition of chymotrypsin-like (ChT-L) and caspase-like (C–L) activities of SDS-activated human 20S ([E] = 2.7 nm) by BPTI and Z-LLL-CHO (A). Inhibition of trypsin-like (T-L) activity of latent human 20S ([E] = 2.7 nm) (**B**). Incubation time was about 30 min. at 37°C.

Data summarized in Table 2 show the inhibitory activities of monocyclic SFTI-1 and its nine analogues against the human and yeast 20S proteasome. The classical aldehyde proteasome inhibitor Z-LLL-CHO was used as the positive control. Since BPTI has previously been described as a strong *in vitro* and *ex vivo* inhibitor of the rat and porcine 20S proteasome [13], its inhibitory activity was also examined (Fig. 2).

**Table 2:** Inhibitory activities of monocyclic SFTI-1(**I**) and its analogues at pH 8.1 and 37°C.

	ChT-like					T-like		C-like	
	Yeast 20S		Human 20S			Human 20S		Human 20S	
	IC_50_	K_a_×10^5^	IC_50_	K_i_	K_a_×10^5^	IC_50_	K_a_×10^5^	IC_50_	K_a_×10^5^
Analogue (No.)	[µm]	[m ^−1^]	[µm]	[µm]	[m ^−1^]	[µm]	[m ^−1^]	[µm]	[m ^−1^]
SFTI-1	51.96	N/D	51.58	26.98*	N/D	>100	N/D	58.19	N/D
(**I)**	±4.90		±5.22					±10.26	
[Leu^7^,Lys^8^]SFTI	2.15	5.24	2.66	1.23*	3.94	>100	N/D	6.74	1.23
(**II)**	±0.10	±2.71	±0.17		±0.99			±0.37	±0.42
[Arg^5^,Leu^7^,Lys^8^]	**1.07**	10.46	3.36	1.76*	3.74	>100	N/D	2.42	3.99
SFTI (**III)**	±0.01	±4.72	±0.29		±1.18			±0.04	±1.49
[Arg^7^,Ile^8^]SFTI	5.36	1.42	2.49	1.31*	5.00	60.5	N/D	2.44	3.96
(**IV**)	±0.51	±0.28	±0.16		±2.05	±2.2		±0.43	±1.04
[Arg^5^,Lys^7,8^]	**1.23**	7.86	**0.94**	0.31	10.14	72.16	N/D	**0.61**	15.59
SFTI (**V**)	±0.06	±2.20	±0.07	±0.04	±2.36	±7.72		±0.04	±1.39
[Tyr^5^,Lys^7,8^]	6.60	N/D	2,78	1.43*	3.76	93.08	N/D	4.02	2.50
SFTI (**VI)**	±0.26		±0.63		±0.68	±17.56		±0.36	±0.58
[Phe^5^,Lys^7,8^]	5.34	2.61	3.41	1.75*	3.30	**45.27**	N/D	2.80	3.17
SFTI (**VII)**	±0.38	±1.16	±0.21		±1.27	±6.93		±0.28	±1.50
[Ala^5^,Lys^7,8^]	8.82	1.66	5.80	2.99*	1.49	>100	N/D	5.37	1.64
SFTI (**VIII)**	±0.19	±0.79	±2.05		±0.53			±0.17	±0.19
[Ala^7,8^]SFTI	>100	N/D	>100	N/D	N/D	**62.13**	N/D	>100	N/D
(**IX)**						±13.51			
[Lys^7,8^]SFTI	16.66	N/D	**1.28**	0.66*	7.05	86.66	N/D	**1.12**	6.15
(**X)**	±0.79		±0.09		±2.04	±18.82		±0.03	±2.29
Z-LLL-CHO	**0.26**	38.9	**0.17**	0.10	65.4	48.22	N/D	5.62	1.82
	±0.02	±1.2	±0.01	±0.02	±7.1	±8.30		±0.10	±0.09

*- the K_i_ values were calculated using the online IC_50_-to-*K_i_* converter tool assuming competitive inhibition (BotDB Database [22]); N/D – not determined (very low activity). The highest activities (the lowest IC_50_ values) are in bold. Z-LLL-CHO was used as the positive control (mean±S.E.M., n = 3).

As mentioned previously, BPTI entered the chamber of the 20S core particle, where it interacted competitively and stoichiometrically with all active subunits [13]. Its inhibitory activity against the C-L site was higher (IC_50_ 0.53 µm) than in the case of two standard inhibitors, such as reversible Z-LLL-CHO (1.7 µm) and irreversible epoxomicin (3.6 µm). It has also been reported that BPTI is a better inhibitor of T-L (IC_50_ 0.79 µm) and ChT-L (0.42 µm) activities than a peptide with an aldehyde group (2.3 and 0.78 µm, regarding T-L and ChT-L activities respectively), but that it is weaker than epoxomicin (0.32 and 0.14 µm, respectively) [13]. Initially, we decided to verify the inhibitory activity of BPTI against the human SDS-activated 20S proteasome (Fig. 2). It turned out that BPTI reduced the C-L activity significantly at concentrations lower than 1 µm. This effect was much more pronounced than in the case of the aldehyde Z-LLL-CHO (Fig. 2A). However, the maximal inhibition caused by BPTI did not exceed 80%, even at a concentration of 12 µm (data not shown). For comparison, 2 µm of BPTI was sufficient to inhibit 86% of the porcine 20S C–L activity [13]. In our study, Z-LLL-CHO at a concentration of 4 µm completely abolished the ChT-L activity, while the same amount of BPTI led to a 60% inhibition (Fig. 2A). A ten-fold higher BPTI concentration resulted in an 80% inhibition of the ChT-L site (data not shown).

Z-LLL-CHO and BPTI reduced the T-L activity of human 20S at higher concentrations (Fig. 2B) than in the cited study [13], where the IC_50_ values were 2.3 µm and 0.79 µm, respectively.

### Inhibition of Human 20S ChT-L Activity

**Figure 3:**
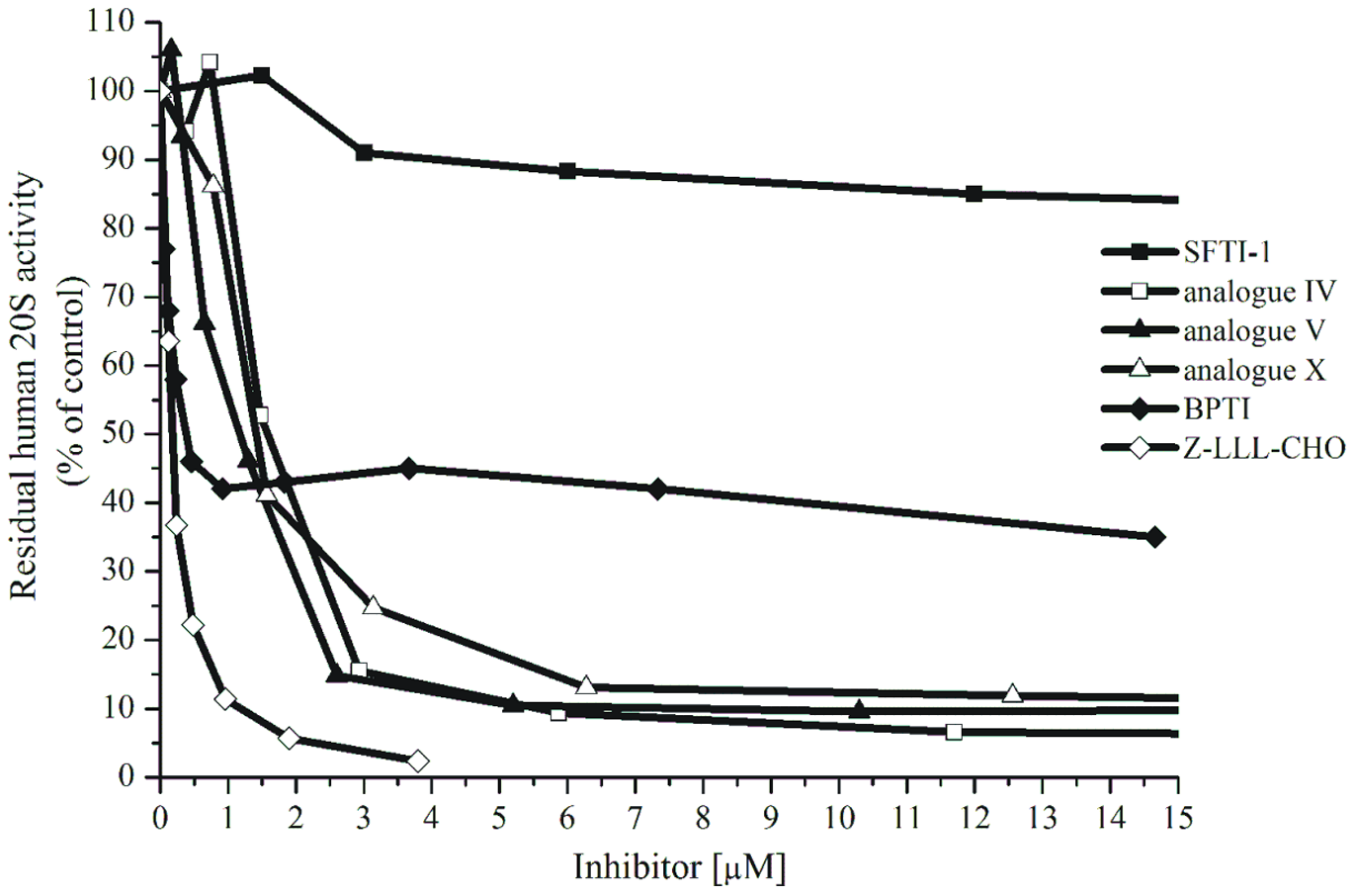
Inhibition of human 20S ChT-L activity by the selected peptides, [E] = 4.4 nm (0.5 µg/200 µL); [S] = 52.4 µm.

Analogue **V** ([Arg^5^,Lys^7,8^]SFTI-1) showed the most significant inhibition of ChT-L human 20S activity (IC_50_ 0.94 µm, Table 2, Fig. 3). This value was almost 55-fold lower than that of the unmodified monocyclic SFTI-1 (IC_50_ 51.6 µm). Peptide **V** contained four positively charged amino acid residues in the sequence. It differed from the parental SFTI-1 (two positively charged residues) in three positions: P_1_ (Arg instead of Lys), P_2_′ and P_3_′ (Lys residues instead of Ile and Pro). Further examination indicated that its K_i_ value was only about 3 times higher (K_i_ 0.31 µm) than that of Z-LLL-CHO (K_i_ 0.1 µm).

**Figure 4:**
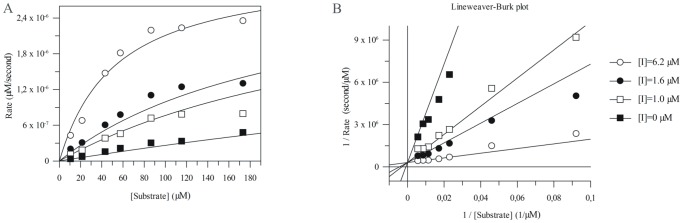
The rate of substrate Suc-LLVY-AMC hydrolysis (a release of AMC molecule) with control and inhibitor V- treated human 20S versus the substrate concentration (A), the enzyme concentration was kept constant at 2.7 nm; the double reciprocal Lineweaver-Burk plot (B).

According to the Lineweaver-Burk double reciprocal plot, the inhibition was competitive (Fig. 4). The association constant value of analogue **V** (K_a_ = 1.0×10^6^ M^−1^, Table 2) was about 6.5 times lower than that of Z-LLL-CHO (K_a_ = 6.5×10^6^ M^−1^). It is worth noting that K_a_ values determined previously in our laboratory, either for monocyclic SFTI-1 and bovine *β*-trypsin or for modified SFTI-1 ([Phe^5^]SFTI-1) and bovine *α*-chymotrypsin, were almost 4 orders of magnitude higher, 9.9×10^9^ M^−1^ and 2.0×10^9^ M^−1^, respectively [10]. The direct comparison of these K_a_ values could indicate that the analyzed interactions between SFTI-1 analogues and human 20S proteasome were significantly weaker. On the other hand, this could be due to the easier access of the inhibitor to the serine protease active site, which is typically located on the enzyme surface. The remaining peptides, with the exception of the peptide **IX** ([Ala^7,8^]SFTI-1), also inhibited the ChT-L activity better than the unmodified SFTI-1 (Table 2). All of them had at least three positively charged residues and at least one (Lys or Arg) in the P_2_′ or P_3_′ position (Fig. 1).

### Inhibition of Yeast 20S ChT-L Activity

All peptides were analyzed against the SDS-activated yeast 20S proteasome ([E] = 0.9 nm Table 2). The highest inhibitory activity was observed for analogues **III** ([Arg^5^,Leu^7^,Lys^8^]SFTI-1, IC_50_ 1.07 µm) and **V** (IC_50_ 1.23 µm). Both IC_50_ values were around 50-fold lower than that of monocyclic SFTI-1 (IC_50_ 52 µm), but higher than that of Z-LLL-CHO (0.26 µm). Compound **IX** with two Ala residues in P_2_′ and P_3_′ had the lowest inhibitory activity among the inhibitors tested. Even at a concentration of about 100 µm, this compound did not reduce ChT-L activity to less than 70%.

It was also interesting that the IC_50_ value of analogue **X** ([Lys^7,8^]SFTI-1) was about 14-times higher (16.7 µm) than that of analogue **V** ([Arg^5^,Lys^7,8^]SFTI-1, 1.2 µm). Both peptides contained four positively charge residues, and differed only in the single P_1_ position – peptide **V** had Arg, while analogue **X** had a Lys residue. It seems that, in addition to the presence of the positively charged residues in P_2_′ and/or P_3_′ positions, Arg in P_1_ position is necessary to obtain a strong proteasome inhibitor.

**Figure 5:**
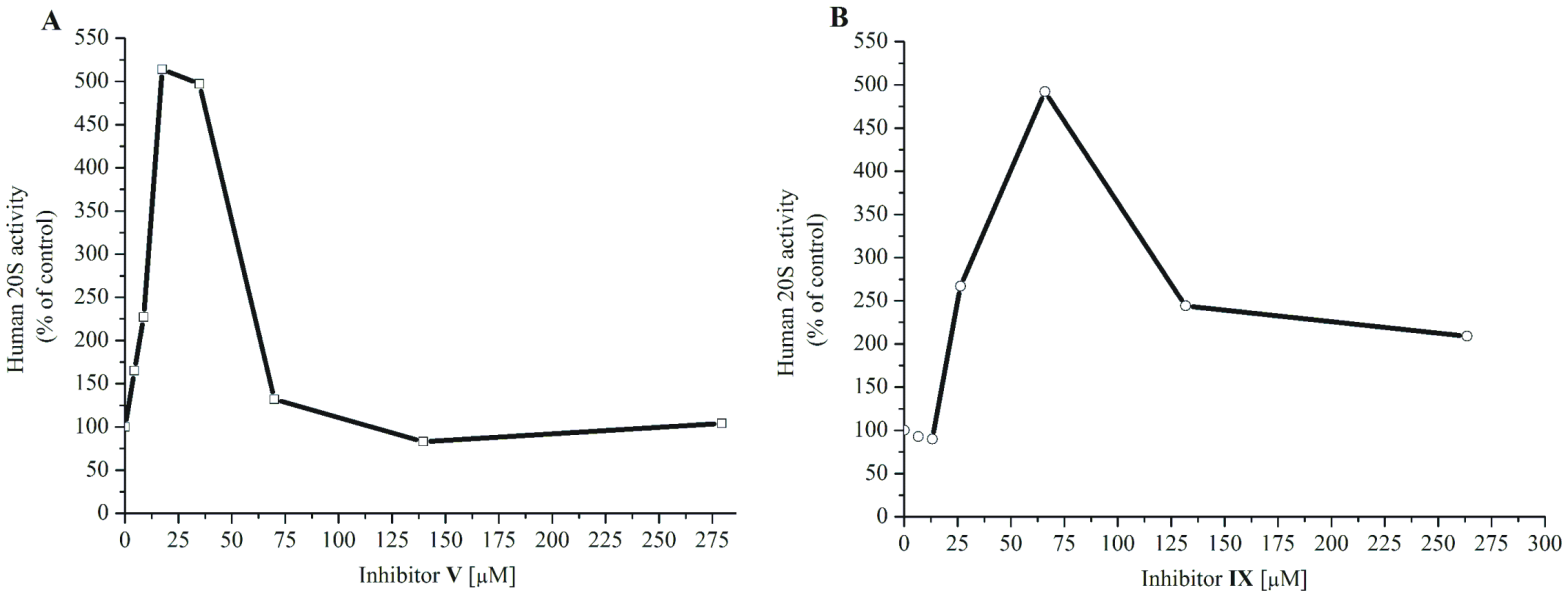
Assays in 50m Tris-HCl buffer without SDS (A) analogue V; (B) analogue IX. Both peptides stimulate ChT-L activity of latent yeast 20S proteasome; [E] = 0.9 nm; [Suc-LLVY-AMC] = 5.2 µm; incubation time 30 min. at 37°C. Data for analogue III are not presented.

Peptide **X** was not as potent inhibitor of the yeast ChT-L activity (IC_50_ about 16.66 µm) as in the case of the human ChT-L site (IC_50_ about 1.28 µm). Considering all the peptides tested, this was the most significant difference between enzymes derived from distinct species. Analogue differences have been previously reported in the literature [24], [25]. Interestingly, it should be emphasized that analogues **III**, **V** and **IX** up-regulated exclusively (about 5-fold) the ChT-L activity of latent yeast 20S proteasome (Fig. 5). This phenomenon was not observed in the assay with the human 20S proteasome.

### Inhibition of Human 20S C-L Activity

**Figure 6:**
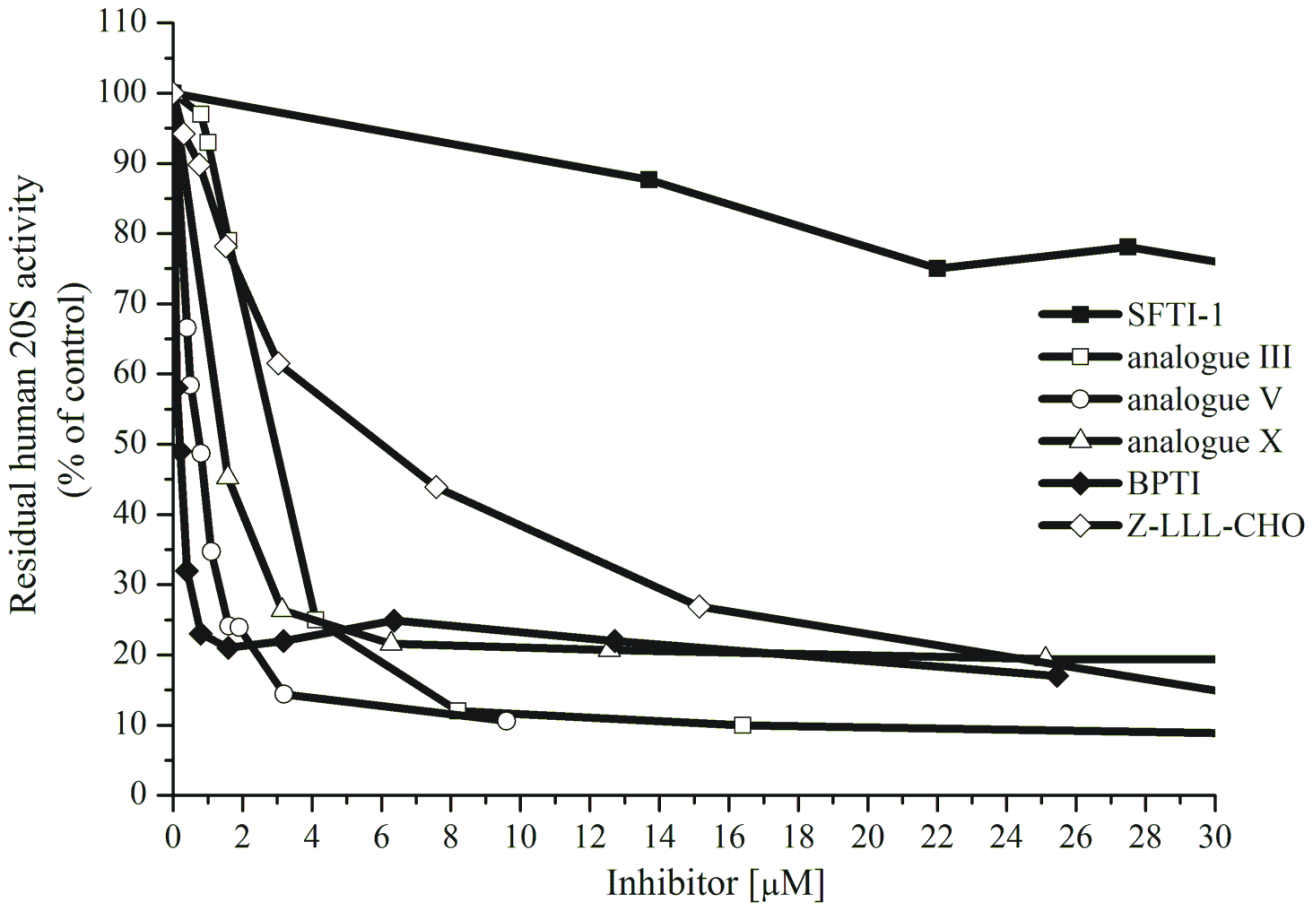
Inhibition of human 20S C-L activity by Z-LLL-CHO, BPTI monocyclic SFTI-1 and its analogues ([E] = 2.7 nm; [S] = 80 µm).

Based on research into tetrapeptides with a *C*-terminal epoxyketone moiety [26], the C-L active site must be considered a co-target along with ChT-L for potential antineoplastic drugs. Analogue **V** showed the highest *in vitro* activity against SDS-activated human 20S (IC_50_ 0.61 µm, Table 2 and Fig. 6), while SFTI-1 was again the weakest inhibitor (IC_50_ 58.2 µm). High activity was presented by analogue **X** (IC_50_ 1.12 µm). The IC_50_ values of analogues **III** and **IV** were around 2.4 µm, this was about 2.3-fold lower than that of the aldehyde based peptide Z-LLL-CHO. In contrast to the ChT-L activity (Fig. 5), the analogues tested were not able to reduce or increase the C-L activity of latent human 20S (data not shown).

### Inhibition of Human 20S T-L Activity

**Figure 7:**
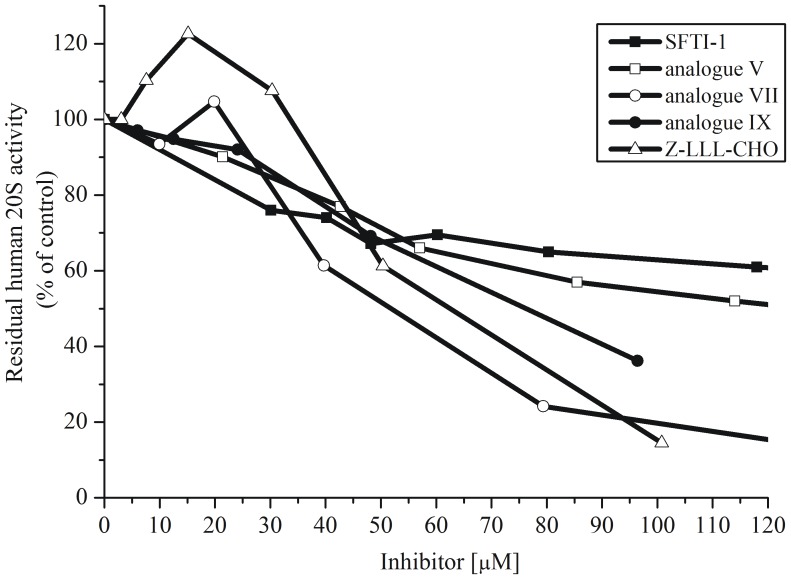
Inhibition of T-L activity of latent human 20S proteasome by Z-LLL-CHO, monocyclic SFTI-1 and its analogues ([E] = 2.7 nm; [S] = 52 µm).

As shown in Figure 7, the peptides displayed weak activity against the T-L site of latent human 20S. Only two IC_50_ values obtained for compounds **VII** and **IX** were around 50 µm. For comparison, BPTI was a somewhat stronger inhibitor reducing 60% of the T-L activity at a concentration of around 40 µm (Fig. 2B). It is likely that the lack of SDS in the Tris buffer makes peptides difficult for entry into the 20S chamber and subsequent interaction with the *β*2 subunit. Additionally, the peptides did not induce the T-L site activation of latent human 20S particle.

### Proteolytic Stability of SFTI Analogues

**Figure 8:**
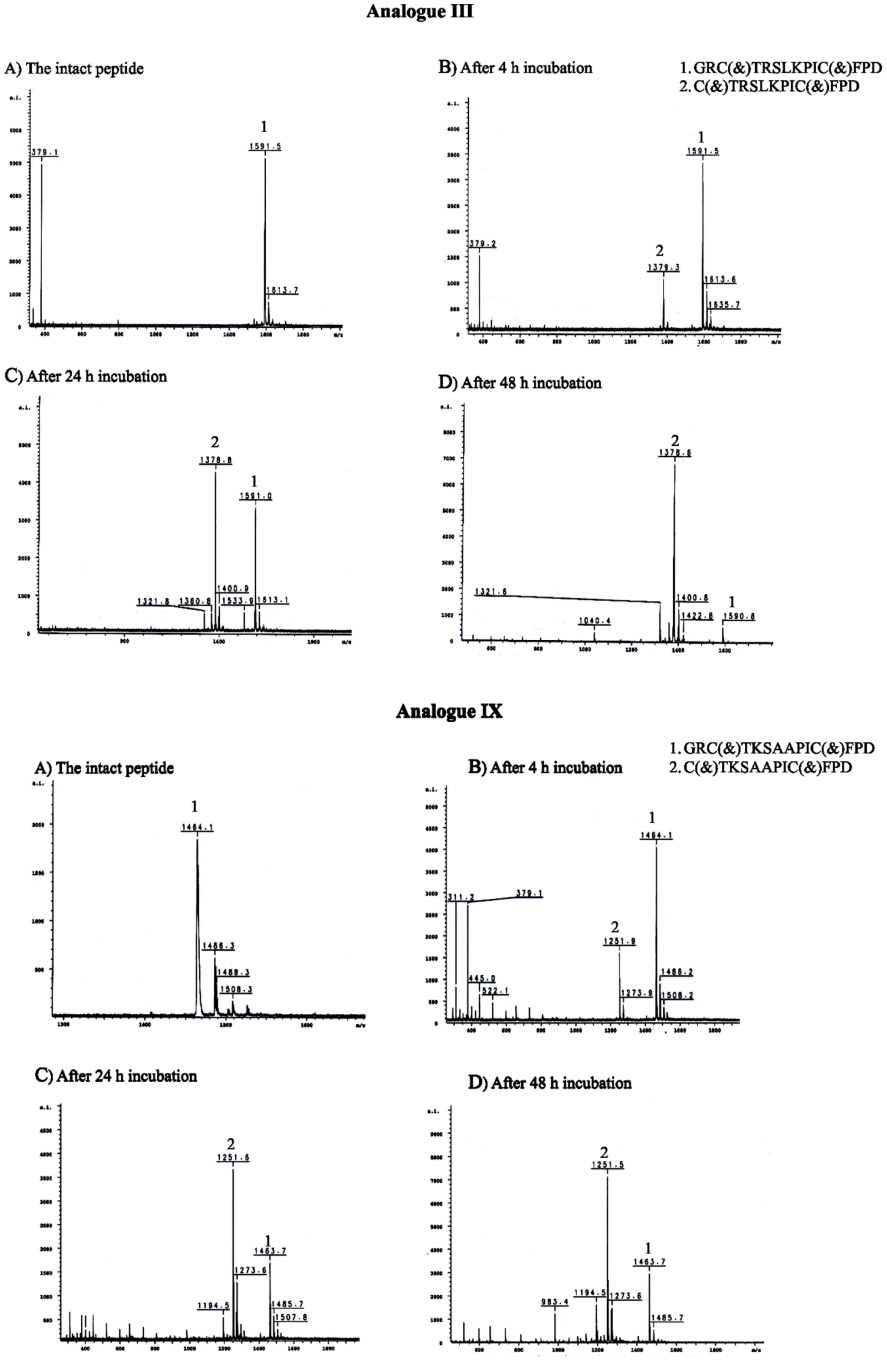
Mass spectra of analogue III (M.W = 1590.9 Da; Cm = 103.0 µm) and IX (M.W = 1463.7 Da; Cm = 134.0 µm) recorded before incubation and after 4 hours, 24 hours and 48 hours incubation with SDS-activated yeast 20S proteasome ([E] = 0.18 µm).

Based on MALDI-MS and RP-HPLC analyses (data not shown), analogues **I**–**V** were not degraded by the SDS-activated human 20S proteasome, even after 24 hours incubation. Furthermore, the assay with the SDS-activated yeast 20S proteasome revealed that analogues **III**, **V** and **IX** were degraded in a similar manner (Fig. 8). The MS signal (*m/z* 1379 and 1252) corresponded to truncated peptides **III** and **IX**, respectively. The truncated peptides were deprived of the *N*-terminal dipeptide Gly-Arg. The intensities of both signals increased with time. This experiment suggested that the analogues entered the yeast 20S interior and interacted with the active subunits.

## Discussion

Proteasome inhibitors are mostly short peptide-based electrophiles that interact in the catalytic subunit with *N*-terminal Thr residues to form a covalent adduct. Among them, peptide aldehydes, vinyl sulfones, epoxyketones, peptide boronates as well as *β*-lactones constitute the well-identified and widely explored groups [3]. Compared to normal cells, cancer cells are much more prone to apoptosis triggered by inhibition of proteasomes. This explains the unquestionable success of the reversible dipeptidyl boronic acid (bortezomib) approved for treatment of relapsed and refractory multiple myeloma and refractory mantle cell lymphoma [4], [5]. However, covalent inhibitors are mostly highly reactive, unspecific and instable. Moreover, inherent or acquired resistance to bortezomib remains a significant threat [27]. Therefore, researches are in progress aimed at developing inhibitors that use different mechanisms than bortezomib [28]. Theoretically, non-covalent inhibitors evoke weaker side effects due to their time-limited, reversible interactions with proteasomes. This less extensively investigated category of inhibitors includes natural cyclic peptides (termed TMC-95A -B,-C and -D) isolated as fermentation products of *Apiospora montagnei* and their mimics [29]–[38]. These peptides contain three amino acids: l-tyrosine, l-asparagine and oxidized l-tryptophan, a biaryl linkage between aromatic side chains and unusual groups at their *N*- and *C*-termini [29], [30]. TMC-95A is the most abundant and the most active diastereoisomer. It competitively inhibits the ChT-L, T-L and C-L activities of 20S proteasome with IC_50_ values of 5.4, 200 and 60 nm, respectively [29]. TMC-95B reduces these activities to the same extent as TMC-95A, while TMC-95C and D are 20–150 times weaker. TMC-95A adopts an antiparallel *β*-sheet structure and binds to the active sites of the proteasome *via* a tight network of hydrogen bonds [30]. TMC-95A shows cytotoxic activities against human cancer cells HCT-116 and HL-60 with IC_50_ values of 4.4 and 9.8 µM, respectively [29]. Further research [32] has indicated that TMC-95A inhibits the ChT-L, T-L and C-L activities of 20S proteasome with K_i_^app^ (apparent K_i_ constant) values of 1.1 nm, 0.81 µm and 29 nm, respectively. Furthermore, less potent simplified cyclic [33], [34], non-constrained linear [35], [36] and dimerized linear mimics of TMC-95A [37], [38] have also been synthesized and analyzed. Blackburn *et al.* [39], [40] screened a library of around 350 000 *C*- and *N*-terminally capped tripeptides derived from the unnatural amino acid *S*-homo-phenylalanine that potently and selectively inhibited the ChT-L activity of the mammalian and yeast 20S proteasomes. The most potent compound demonstrated an IC_50_ value of 1.2 nM for the human 20S *β*5 site *in vitro* and a K_i_ below the enzyme concentration in the assay (0.25 nm 20S) [39]. It is interesting that this compound presented greater affinity for the *β*5 site than the covalent inhibitor bortezomib. Further optimization [40], guided by X-ray crystallography of compounds in complex with the purified yeast 20S, yielded a series of non-covalent di-peptide inhibitors of the proteasome with unprecedented *in vitro* and cellular potencies. The most active inhibitor reduced exclusively the ChT-L activity with IC_50_ = 7.4 nm. Moreover, Furet *et al.* [41], [42] analyzed pseudopeptides such as the 2-aminobenzylstatine derivatives that specifically inhibit the ChT-L site of the human proteasome with an IC_50_ value in the micromolar range. Gallastegui *et al.* [43] presented non-peptidic hydroxyureas (IC_50_ = 300 nm and K_i_ = 34 nm for the most active and highly selective inhibitor of the yeast 20S ChT-L site), whereas Formicola *et al.* [44] described novel inhibitors of rabbit 20S proteasome based on the trifluoromethyl-*β*-hydrazino acid scaffold, with differential inhibitory capacity for ChT-L, T-L and C-L in the micromolar range. Thus far, there have not been many reports describing inhibitory activity against proteasomes presented by the proteinaceous inhibitors of serine proteases. Here, we have presented that SFTI-1, although a weak inhibitor of the yeast and human 20S proteasomes, can be successfully used to design much more potent inhibitors. Peptide V inhibited the ChT-L and C-L activities of yeast and human 20S proteasome with IC_50_ values of 1.2 µm, 0.9 µm and 0.6 µm, respectively (Table 2). We have confirmed that the presence of at least one basic amino acid residue (Lys or/and Arg) in the position P_2_′ or/and P_3_′ is of significance to obtain potent inhibitors. Additionally, comparing the activity of peptide **V** and **X** against the yeast 20S proteasome, we proved that the type of amino acid residue in P_1_ position is also important. Peptide **V** with Arg residue was a better inhibitor of the ChT-L activity than peptide **X** with Lys. Moreover, we provided evidence that the peptides enter the 20S chamber. Some of the analogues underwent partial degradation when incubated with SDS-activated yeast 20S proteasome. The competitive mode of inhibition resembles the interaction between BPTI and rat 20S proteasome [13]. Unfortunately, an X-ray crystal structure analysis of a putative complex between the yeast 20S proteasome and analogues **V** or **III**, at a resolution of 3.1 Å, did not reveal any electron density related to the peptides (data not shown).

Interestingly, peptides **III**, **V** and **IX** stimulated exclusively the ChT-L activity of latent yeast 20S proteasome. A similar up-regulation caused by an HIV-1 Tat derived peptide was observed by Jankowska *et al.* [45]. The authors postulated that this resulted from electrostatic interactions between the highly positively charged peptide and acidic patches found on the surface of 20S particles. It is likely that SFTI-1 analogues may also adhere, *via* their basic residues, to the outer surface of yeast 20S *α*-subunits and trigger the opening of the latent 20S core particle.

Finally, we identified novel non-covalent inhibitors of human and yeast 20S proteasomes and provided evidence that SFTI-1 can be used as a template for preparation of potent 20S proteasome inhibitors.
